# Abdominal Cocoon Syndrome: An Extremely Rare Cause of Small Bowel Obstruction

**DOI:** 10.7759/cureus.14351

**Published:** 2021-04-07

**Authors:** Paraskevi Karona, Evangelos Blevrakis, Pagona Kastanaki, Aggelos Tzouganakis, Miltiades Kastanakis

**Affiliations:** 1 First Department of Surgery, Saint George General Hospital of Chania, Chania, GRC; 2 Pediatric Surgery Department, Saint George General Hospital of Chania, Chania, GRC

**Keywords:** cocoon syndrome, intestinal obstruction

## Abstract

Abdominal cocoon syndrome or encapsulating peritoneal sclerosis is a rare condition causing small bowel obstruction. It is called cocoon syndrome because of the existence of an abnormal membrane that contains part or the entire small intestine. We present a case of a 49-year-old male, presenting to our department with recurrent episodes of obstructive ileus that did not respond to conservative treatment. He underwent exploratory laparotomy and a thick membrane covering the small bowel loops was found. The membrane was excised and sent for pathological examination. Abdominal cocoon syndrome is an acquired condition caused by an inflammatory process that is not yet completely understood. There are many theories for the pathophysiology of the disease. In most cases, the diagnosis is established during surgery. Complete removal of the membrane is the indicated surgical treatment. In mild cases, when the diagnosis is made preoperatively, conservative treatment should be the first choice.

## Introduction

There are reports in the literature about the existence of an abnormal membrane encapsulating the intestine since 1968 [1]. There are several different presentations of this abnormality [1]. Abdominal cocoon syndrome also known as primary sclerosing encapsulating peritonitis is a rare cause of small bowel obstruction [2]. The exact pathophysiology of the disease remains unknown, although many hypotheses have been made [2,3]. Computed tomography with intravenous contrast is considered the most useful radiological examination for the diagnosis of this abnormality as far as for the decision-making [4]. In most cases, the definitive diagnosis is made during surgery [2,3].

## Case presentation

A 49-year-old male experienced recurrent obstructive ileus, counting two admissions in our department within three months, treated conservatively. The third time he underwent an exploratory laparotomy when all conservative measures failed.

The patient's previous medical history included only hyperlipidemia, treated with an oral agent. One month before the first episode of intestinal obstruction he underwent emergency surgery in another institution for acute appendicitis and appendectomy was performed. The surgeon noticed an abnormal membrane covering part of the ileus, and a sample of the tissue was sent for histological examination. The findings were non-specific, indicating a membrane formed of connective tissue.

**Figure 1:**
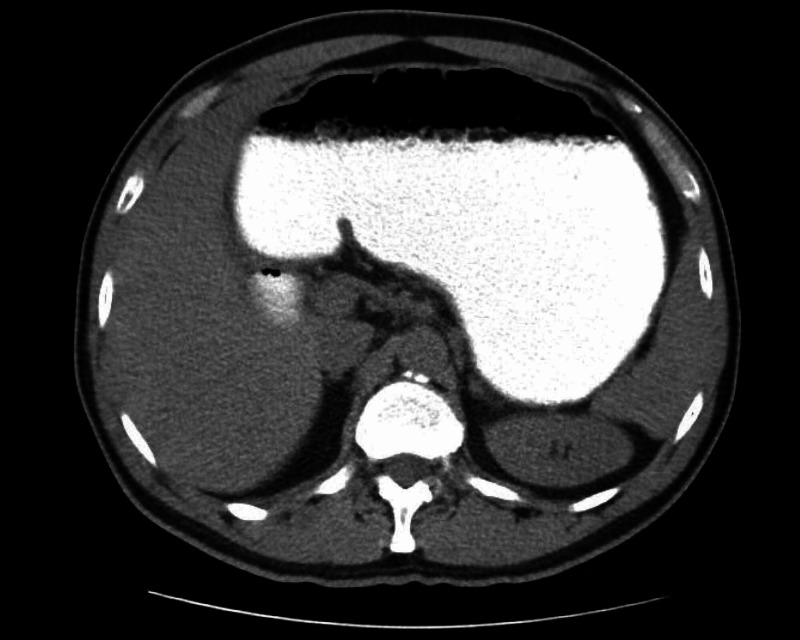
CT scan. CT scan after gastrografin administration revealing distended stomach.

**Figure 2:**
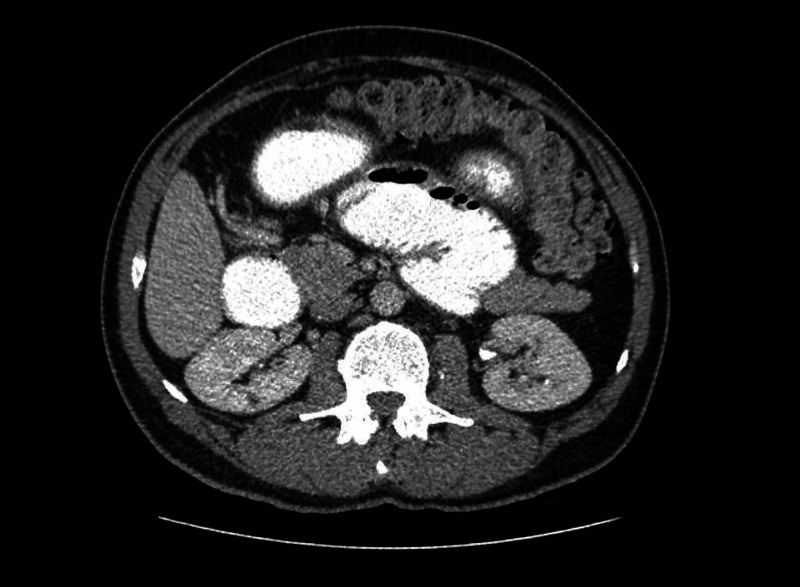
CT scan. CT scan after gastrografin administration revealing distended small bowel loops.

The diagnostic workup during his second admission included an upper GI endoscopy and a barium-contrast examination. A CT scan with oral and intravenous contrast was scheduled in the last admission. The endoscopy revealed gastritis, caused by helicobacter pylori, while the barium study showed no abnormalities. The CT scan revealed a distended stomach and upper jejunum, especially near the ligament of Treitz (Figures 1, 2). It also revealed an area of the jejunum with thickened wall and narrowed lumen. 

The patient experienced incapacity of feeding properly and was malnourished. During hospitalization he was supported with parenteral nutrition and a decision of laparotomy was made, as no amelioration of his condition was observed with conservative measures.

**Figure 3:**
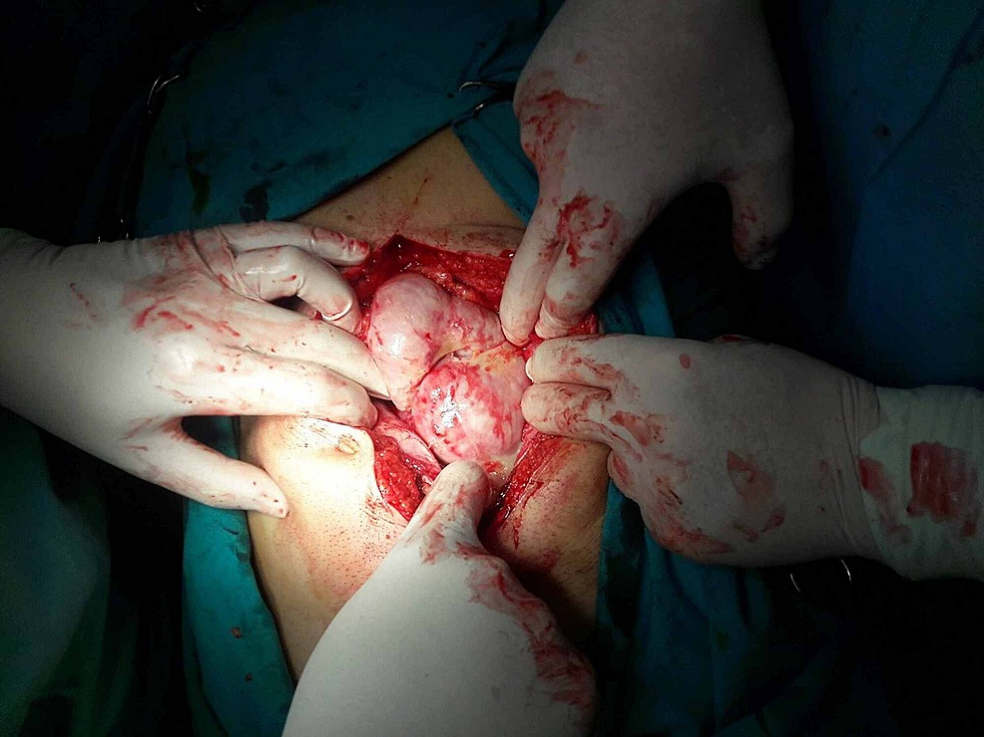
Intraoperative findings. By entering the abdominal cavity a thick membrane covering the small intestine is identified.

**Figure 4:**
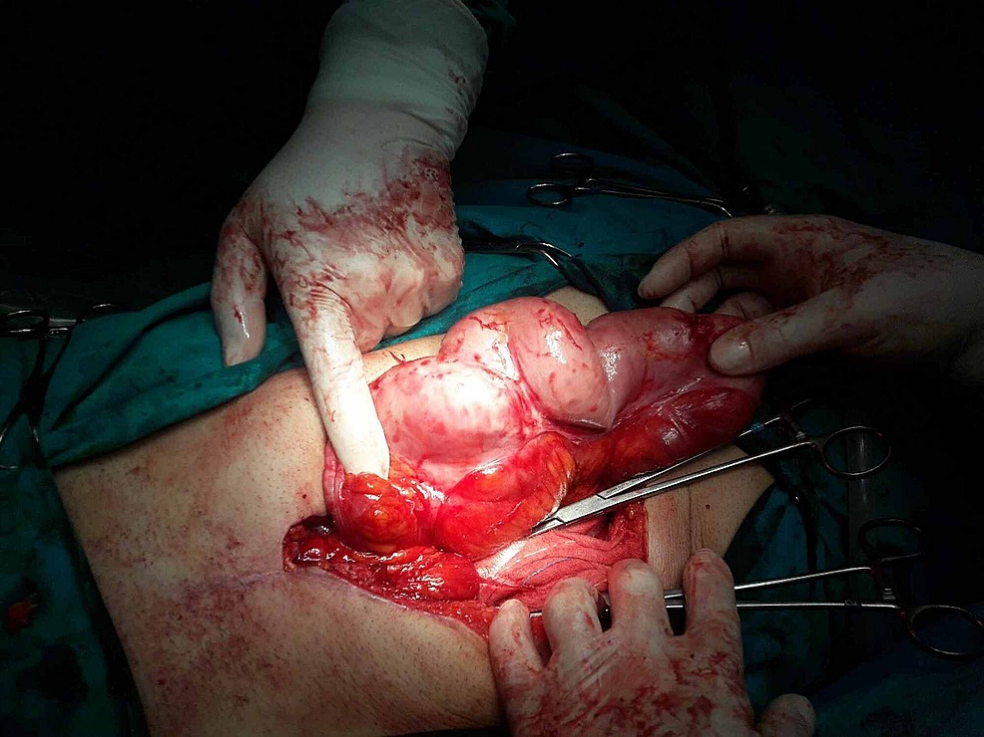
Intraoperative findings. A loop completely covered with the membrane.

**Figure 5:**
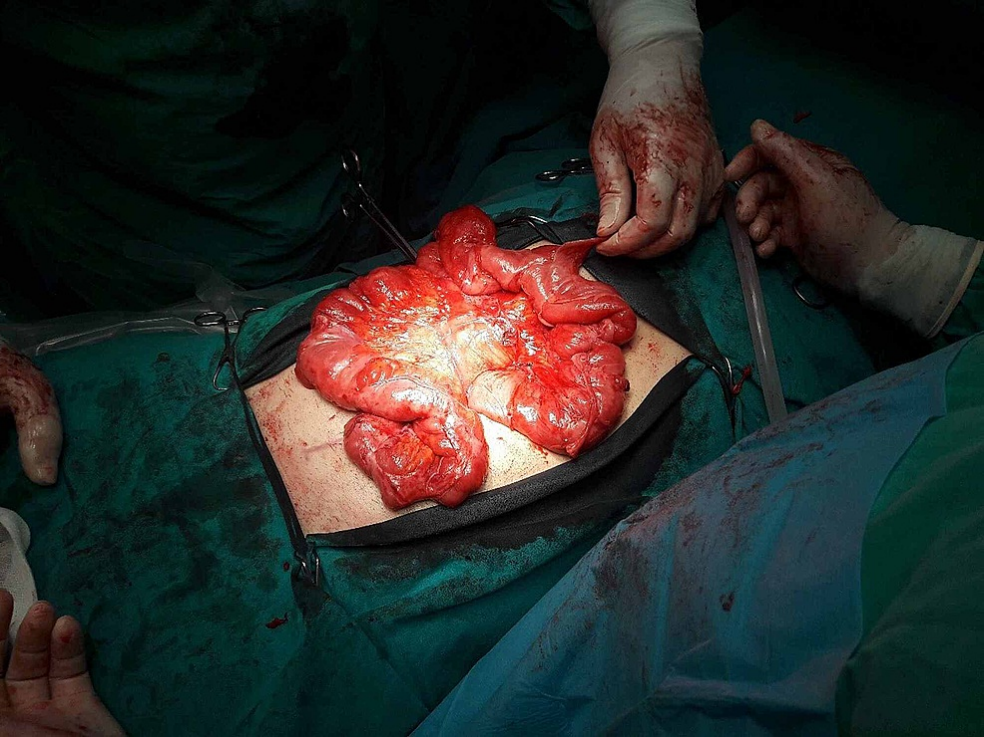
Intraoperative findings. The membrane is completely removed.

He underwent laparotomy through a midline incision. By entering the abdominal cavity, a dense membrane was found encapsulating the stomach, the small intestine and part of the large intestine (transverse colon and the front of the ascending and descending colon) (Figures 3, 4). The whole membrane was excised and extended adhesiolysis was performed, releasing the small bowel loops (Figure 5). The entire small intestine was viable, without serosal tears. The patient had an uncomplicated postoperative period and left hospital the 12th postoperative day. 

The membrane was sent for histological examination. The pathology report of the membrane revealed connective tissue with the presence of inflammatory cells.

One week after he was discharged, the patient experienced retching and vomiting when consuming liquids, while surprisingly he could tolerate eating solid food. He was advised to be hospitalized again with food restriction and Levin catheter. There was a high suspicion of cocoon syndrome and the histological results would confirm the diagnosis. He denied admission and visited another institution, where he underwent another laparotomy.

## Discussion

The existence of an abnormal membrane encapsulating the intestine has been reported in the literature since 1968 [1]. There are several different variations of this abnormality [1]. In 1968, Cleland described a disease with the name peritoneal encapsulation [1,2,4]. He reported the presence of an additional peritoneal membrane, which is supposed to be derived from the yolk sac peritoneum [4]. There is no evidence that this membrane is formed due to an inflammatory process, so the disease is considered congenital [4]. This membrane usually covers the small intestine and has similar composition and structure with the peritoneum. [1] In most cases, this condition remains asymptomatic [1].

One could suppose that this disease is a variation of the well-known retroperitoneal fibrosis, known also as Ormond disease [5]. In retroperitoneal fibrosis, the presence of fibrosclerotic alteration in the retroperitoneum often causes encasement of the ureters [5].

Most cases referred to in the literature with an additional abdominal cavity membrane are supposed to be related to an inflammatory process. Encapsulating peritoneal sclerosis (also known as sclerosing encapsulating peritonitis [SEP]) is an acquired condition [4]. The main difference with peritoneal encapsulation is histopathological. In the first case, the membrane is composed of connective tissue with inflammatory cells [1,4]. The disease is either primary or secondary.

Primary SEP is also named idiopathic SEP or cocoon syndrome. The term cocoon syndrome was first introduced by Foo in 1978 [4-6]. The cause of the disease remains unknown. It is considered to affect mostly males [1]. There are also reports of young females in tropical countries, suffering from primary SEP [1,4-7].

Abdominal cocoon syndrome is subcategorized into three types. In the first type, only part of the small intestine is encased. In type 2, the membrane covers the entire small intestine, while in type 3, other organs, such as part of the colon, ovaries, liver, stomach, or the appendix are also included in the encapsulated viscera [1,2,4].

Secondary SEP is highly associated with peritoneal dialysis, intra-abdominal inflammation, previous abdominal surgery or trauma and beta-blocker intake [2,3,6,7].

The case we present is believed to have primary sclerosing encapsulating peritonitis type 3, as the membrane existed in the first surgery and part of the stomach and large bowel were also encased.

The most common manifestation of this rare syndrome is intestinal obstruction [4,7]. In the majority of cases, preoperative diagnosis is not specific and the disease is confirmed during surgery. The presentation varies and can be acute, subacute or chronic [2,6-8]. Other symptoms include abdominal discomfort, tenderness, nausea and intestinal distention [2,4,6,8]. In the chronic form patients suffer from weight loss and nutritional abnormalities [2,4].

A preoperative diagnosis is not usually established. Most patients undergo exploratory laparotomy for intestinal obstruction. Plain abdominal X-rays are not specific and they may reveal air-fluid levels, which are indicative of intestinal obstruction. Computed tomography is considered to be more accurate, as it can reveal the concentration of small bowel loops in the central part of the peritoneal cavity and the presence of a thick membrane encapsulating bowel loops [1,4,6,7]. A thinner membrane may not be seen [7]. Although in most cases the diagnosis is not established preoperatively, CT is considered to be the gold standard [3,7].

Therapeutic options vary from conservative management in mild cases to surgical intervention, open or more recently laparoscopic, which is the most common [1,7,8]. Conservative management includes nil per mouth, nasogastric tube and enteral or parenteral nutrition [1,4,7]. Patient showing amelioration with this approach could be treated in a chronic basis with medications, such as colchicine, steroids and immunosuppressants [5,7].

When complete intestinal obstruction is the case, surgery is the only choice of treatment [7]. When surgery is performed, the entire membrane encapsulating the small intestine should be removed, although it could cause an obstructive ileus in the early postoperative period [2,4].

## Conclusions

Abdominal cocoon syndrome is a rare cause of intestinal obstruction. Surgeons should consider this abnormality as a possible cause of chronic abdominal pain and bowel obstruction when other common causes are excluded. Contrast-enhanced abdominal CT scan seems to be the better diagnostic method, although in most cases the diagnosis is established in the operating room.
